# Differences in habenula and septal nuclei and resting state functional connectivity (RSFC) in the presence or absence of clinically significant insomnia in patients with MDD

**DOI:** 10.1192/j.eurpsy.2024.810

**Published:** 2024-08-27

**Authors:** J.-M. Heo, S.-G. Kang, S.-E. Cho, C.-K. Kang, J.-Y. Jung

**Affiliations:** ^1^Psychiatry, Gil Medical Center, Gachon University College of Medicine; ^2^Health Science, Gachon University Graduate School; ^3^Radiological Science, College of Health Science, Gachon University, Incheon, Korea, Republic Of

## Abstract

**Introduction:**

There are differences in clinical presentation with and without insomnia in MDD, and it is expected that there are brain biological differences that contribute to this, but functional MRI studies of MDD with insomnia vs MDD without insomnia are scarce. In particular, few studies have examined resting state functional connectivity (RSFC) seeding the habenula and septal nuclei, which play key roles in both mood and sleep.

**Objectives:**

The purpose of this study is to determine whether there are differences in habenula and septal nuclei and RSFC in the presence or absence of clinically significant insomnia in patients with MDD.

**Methods:**

To identify the effects of insomnia in MDD group, one-way ANCOVA covariate control was used to compare differences of RSFC between MDD_w/INS and MDD_wo/INS group. The potential confounders (i.e., age, sex, education years, and total score of HDRS-17) were adjusted in this analysis. To examine the relationship between RSFC and clinical sleep questionnaires (i.e., ISI and PSQI) in the participants with MDD, Pearson’s partial correlation analysis controlling same potential confounders was performed by using Fisher-transformed correlation coefficients and scores of ISI and PSQI. For comparing the difference of RSFC between MDD and HC, the analysis was also performed with ANCOVA controlling for age, sex, education years.

**Results:**

The analysis in this study included 36 in the MDD_w/INS group, 21 participants in the MDD_wo/INS group, and 38 in the healthy controls (HC) group. The main finding of this study was that MDD with insomnia showed increased RSFC in Habe_L - Rolandic_Oper_R, Habe_L - Cuneus_R, Habe_R - Thal_Pul_R, and decreased RSFC in Septal - Cerebellum_Crus1_R compared to MDD without insomnia. All regions with significant results were significantly correlated with insomnia severity.

**Conclusions:**

Since the RSFC of all pairs of regions that showed significant differences between the two groups in this study were significantly correlated with insomnia severity (i.e., ISI score), the association of these regions with insomnia in MDD is supported. The significance of this study is that there have been studies that have examined the RSFC in fMRI for insomnia, but there are few studies on MDD with insomnia, and since the habenula and septal nuclei play an important role in insomnia, sleep, and mood, it is meaningful to seed fMRI studies on these areas.

**Disclosure of Interest:**

None Declared

